# Far-field mapping and efficient beaming of second harmonic by a plasmonic metagrating

**DOI:** 10.1515/nanoph-2023-0842

**Published:** 2024-04-17

**Authors:** Augustin Verneuil, Agostino Di Francescantonio, Attilio Zilli, Julien Proust, Jérémie Béal, Daniela Petti, Marco Finazzi, Michele Celebrano, Anne-Laure Baudrion

**Affiliations:** Light, Nanomaterials, Nanotechnologies, 27093 Université de Technologie de Troyes, Troyes, France; Dipartimento di Fisica, 18981 Politecnico di Milano, Milan, Italy

**Keywords:** metasurface, nonlinear optics, second-harmonic generation, Fourier imaging, plasmonics, gold

## Abstract

We study numerically and experimentally the second-harmonic generation (SHG) from rectangular metagratings of V-shaped gold nanoantennas. We show that by carefully engineering the array pitch to steer the diffraction orders toward the single antenna emission, the extracted signal is maximized. This enhancement is attributed to the angular overlap between the radiation pattern and array factor and is comparable to the improvement yielded by the coupling of surface lattice resonances (SLRs) with local modes. Moreover, we demonstrate a simple technique to experimentally reconstruct the emission diagram of an antenna from measurements of the collective grating response as a function of the excitation angle. Excellent agreement is obtained with simulations when the sample is immersed either in air or in water, which is crucial in view of future sensing application. Thanks to the high signal-to-noise ratio and low dependence on the statistical particle dispersity, this method constitutes an effective alternative to back-focal plane imaging when very weak signals such as SHG are involved.

## 1 Introduction

In recent years, the development of fabrication techniques capable of nanometer-scale shaping and positioning has ushered in novel classes of materials capable of unprecedented light–matter interactions. Among them, metal nanoantennas became particularly attractive as second-harmonic generation (SHG) sources for a number of reasons. Indeed, in plasmonic metals, even-order nonlinearities are dipole-forbidden due to the inversion symmetry of the crystalline lattice [1], [2], [3]. Hence, the main contribution to SHG stems from nonlinear currents at the metal–environment interface [4], [5], offering a sensitive probe of surface effects. In this context, nanoantennas are especially attractive due to their large surface-to-volume ratio. Moreover, in metal structures, the electric field can be locally increased by the lighting-rod effect. Another appealing feature of metallic nanoparticles is represented by the localized plasmon surface resonance (LSPR) modes that they can support. Local electric fields are in fact enhanced when such resonances are excited, leading to a consequent enhancement of the SHG yield, which scales quadratically with the field intensity [2], [5], [6]. SHG also benefits from the increased local density of states [7]. In fact, the best upconversion rates for metal nanoantennas have been reported for structures supporting two spatially overlapping plasmonic resonances at the fundamental and harmonic wavelengths, thus improving both the absorption of the fundamental wavelength and the emission of the harmonic wavelength [8], [9].

A characteristic limitation of metals is represented by Ohmic losses, which deteriorate the resonance quality factor by damping the electronic resonance. However, surface lattice resonances (SLRs), also known as Rayleigh anomalies, have attracted much attention due to the high quality factor they are associated with. These stem from evanescent waves traveling in the lattice plane that can couple with the LSPR of neighboring antennas, when the two are in phase, alleviating the issue of losses in plasmonic platforms [10]. The spectral position of the SLR is given by the array parameters and surrounding refractive indices [11] and can additionally be controlled using the incident angle [12]. SLRs are associated with improved local field enhancement and narrow spectral linewidth and have spurred intense research and applications in many domains, such as photonics, sensing, photovoltaics, or nonlinear optics [13], [14], [15], [16]. For instance, arrays supporting collective modes at one or both of the fundamental and harmonic wavelengths have been recently demonstrated, improving the nonlinear conversion rate [17], [18], [19].

However, as these configurations employ subwavelength array periods, only the 0th order of diffraction can be collected in the far-field. Since SHG in plasmonic antennas has often a nondipolar origin and, hence, does not radiate broadside, much of the signal is lost. In this paper, we demonstrate a doubling in the collected SHG signal from a periodic grating of plasmonic nanoantennas by designing the grating to effectively overlap the first diffraction order with the emission of the individual antenna. Moreover, we demonstrate that the single nanoantenna SHG emission diagram can be retrieved by ensemble measurements while tuning the angle of the first diffraction order of the grating.

**Figure 1:**
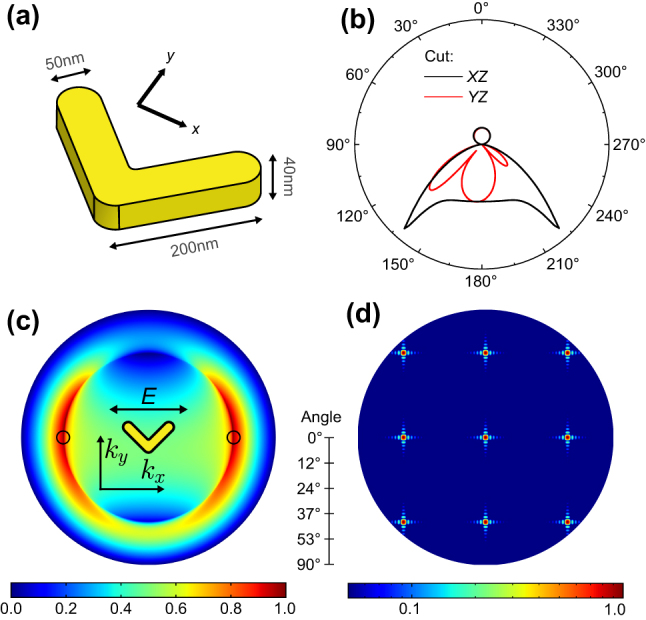
Antenna geometry and calculated far-field-projected SH intensity. (a) Sketch of the nanoparticle and the associated geometrical parameters. (b) and (c) Simulated far-field SHG emission pattern of a single antenna, given as the Poynting vector amplitude normalized to its maximum BFP is not linear in angle and (b) and in the BFP of the substrate half-space (c). The black circles in (c) indicate the position of a diffraction order for the optimized period. (d) Simulated normalized array factor (AF) our grating, for a 25 × 25 configuration, plotted in a normalized logarithmic color scale. The fringes adjacent to the main diffraction orders are caused by the finite number of elements taken into consideration.

Our metasurfaces consist of V-shaped antennas (Figure 1(a)), which exploit a geometry lacking inversion symmetry for efficient SHG emission [3], [20], [21]. Furthermore, this design improves the upconversion rate thanks to two spatially overlapping resonances: one at the telecom fundamental wavelength (FW) *λ*_FW_ = 1550 nm and the tail of another at the second harmonic (SH) *λ*_SH_ = 775 nm [7]. The linear electromagnetic characterization of the antennas in terms of extinction spectra and simulated field maps can be found in the Supplementary Material, in Figures S2 and S4.

## 2 Array optimization for SHG extraction

### 2.1 Numerical simulations

The SH emission behavior was simulated using the commercial finite-elements method (FEM) solver COMSOL, in a two-step scheme commonly employed in the literature [4] (i.e., under the undepleted pump regime). Permittivity data were taken from the literature for gold [22] and from the manufacturer data sheet for the BK7 glass substrate (n_sub_ = 1.51). The field distribution at the fundamental wavelength is first calculated under plane wave illumination at normal incidence (see Figure S1) from the substrate half-space. The nonlinear current sources on the nanoantenna surface are then deduced from the resulting electric field amplitude and used in a second simulation step yielding the field distribution at the harmonic frequency. Finally, we compute the SHG radiation pattern using a near-to-far field transform provided by the RETOP toolbox [23], ensuring a correct projection in the presence of the air–substrate discontinuity.

The resulting emission pattern is given in Figure 1(c), showing a close resemblance to that of an electric dipole oriented along *y*, with some asymmetry due to the dissymmetric shape of the emitter. It features two lobes at 42° from the optical axis, corresponding to the critical angle of an air–glass interface. To obtain a diffraction order in this direction and maximize the SH extraction, we then use Bragg’s law of diffraction gratings *G* = *λ*_SH_/(*n*_sub_ sin*θ*), yielding a pitch *G* = 766 nm.

The grating response can then be synthetized from the single antenna radiation pattern, *f*(*θ, ϕ*). Indeed, in a diffractive grating, the collective far-field response, *F*(*θ, ϕ*), can be described as the sum of each antenna’s angular emission pattern, modulated by a term representing the long-distance interference effects, termed the array factor (AF): $F\left(\theta ,\phi \right)=N\left[f\left(\theta ,\phi \right)AF\left(\theta ,\phi \right)\right]$ where *N* is the number of antennas and (*θ, ϕ*) the polar and azimuthal angular coordinates in the far-field. The emission pattern and array factor are analogous to the form and structure factors found in X-ray diffraction literature, respectively. For a two-dimensional (2D) rectangular grating, the spherical AF can be written as [24], [25]:

(1) $$\begin{array}{ll} AF\left(\theta ,\phi \right) & =\frac{1}{N_{x}N_{y}}\cdot \overset{N_{x}-1}{\underset{n_{x}=0}{\sum }}\overset{N_{y}-1}{\underset{n_{y}=0}{\sum }}\exp ik\left(n_{x}d_{x}\left(\cos\theta \sin\phi +\alpha_{x}\right)\right.\\ & \;\left.+n_{y}d_{y}\left(\sin\theta \sin\phi +\alpha_{y}\right)\right) \end{array}$$

with *N*_*x*_, *N*_*y*_ the number of antennas in the *x* and *y* directions, *k* the wavenumber of the emitted light, *d*_*x*_, *d*_*y*_ the interparticle distance, and *α*_*x*_, *α*_*y*_ the phase delay between individual emitters. This decoupling of the antenna and array responses is valid in the absence of cross-talk between the antennas, i.e., near-field coupling or long distance interactions such as SLRs. Firstly, near-field coupling is possible within the reactive near-field region (*r* < *λ*_SH_/2*π*) [24], which is respected by our array parameters. This is further evidenced by the simulated near-field distributions in Figure S2, showing a rapid decrease of the electric field intensity. Secondly, a detailed analysis of the SLR coupling regime can be found in the Supplementary Material, confirming the validity of the assumption for our experimental parameters.

We calculate the AF for our array configuration and its product with the simulated antenna radiation pattern, yielding the result presented in Figure 1(d).

### 2.2 Experimental details and results

**Figure 2:**
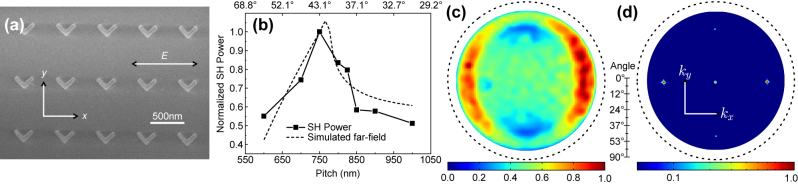
Second harmonic generation form gold nanoantennas. (a) Scanning electron microscopy image of a metasurface, with a period of 600 nm in *x* and 775 nm in *y*. The double arrow indicates the incident polarization for all experiments. (b) Normalized SHG in the (+1;0) diffraction order as a function of pitch in *x*, and the simulated emission pattern, normalized to the data point obtained for a 750 nm pitch. BFP images (c) for a single antenna and (d) for the metasurface with a period in *x* of 800 nm. The data in (c) were smoothed using a Gaussian kernel with a standard deviation of 1.5 pixels. The dashed circles in Figure 2(c) and (d) denote the edge of the Fourier plane, whereas collected data ranges are limited by the objective NA.

We fabricated several arrays, each one with a different periodicity to experimentally verify the optimization, using a standard electron-beam lithography and lift-off process: a layer of resist (polymethylmethacrylate, PMMA), covered by a conductive polymer, is patterned with an electron microscope (eLINE, Raith). After development, an adhesion layer of 4 nm of TiO_2_ is evaporated, followed by 40 nm of gold. Finally, the sample is left in acetone overnight for lift-off. A typical metasurface is shown in Figure 2(a). In addition to the arrays, a few isolated particles are fabricated to study their individual behavior.

The antennas are pumped by a pulsed (160 fs duration) laser at *λ*_FW_ = 1550 nm. The emission pattern of the emitted second-harmonic radiation is collected at the back focal plane (BFP) in a 4*f* arrangement. The complete optical setup is described in Figure S5 of the Supplementary Material. The single emitter’s radiation pattern was first imaged by focusing exciting light on one of the isolated antennas (oil-immersion, with NA = 1.35). Incident power was kept low (100 µW) to avoid photodamage. The measured power distribution in the BFP (Figure 2(c)) is well reproduced by the numerical simulations in Figure 1(c), confirming the location of emission maxima.

The emission of the grating is then characterized in a similar fashion, but with near plane-wave excitation. This is achieved by focusing the laser on the back focal plane of the objective. The excitation spot on the sample is adjusted with a pinhole to be roughly 30 µm in diameter, illuminating the whole grating consisting of approximately 600 antennas. Figure 2(d) is an example of such measurement, and its features are reproduced well by the calculated diffraction pattern in Figure 1, both in terms of position of the diffraction orders and of the relative intensity between (±1; 0) and (0; 0) (2.11 from the product of Figure 1(c) and (d), and 2.01 from numerical simulations).

As the pitch increases along the *x* axis, the diffraction angle decreases, resulting in a different sampling position of the radiation pattern. Thus, we find that the measured intensity (Figure 2(b)) closely follows the behavior of the radiation pattern in the *k*_*x*_ direction, outlined in the previous simulations (Figure 1(b) and (c)). We find that a period of 750 nm, diffracting at 43°, extracts the most SHG, with a factor 2 compared to the value obtained from the array with 600 nm periodicity, despite the latter’s higher density of emitters. This result is in accordance with the simulation and consistent with the idea of an overlap between the single antenna radiation pattern and the diffraction order.

Our findings are in line with previous studies of SHG from V-shaped nanoantennas. It has been demonstrated that closely packing an array of nanostructures can be detrimental to the overall optical response, due to resonance damping caused by near-field coupling [7]. Instead, careful engineering of the array period can result in improved emission despite lower particle density, for instance taking advantage of SLRs [26], or by tuning the diffraction angle as in this work. Moreover, this improvement is comparable to the increase found by Stolt et al. [19] when coupling the LSPR at the harmonic with an SLR.

## 3 Radiation pattern reconstruction

As the collected signal results from the single nanoantenna SHG emission sampled by a diffraction order, the former can be reconstructed by sweeping the position of the diffraction order in the BFP. In the array factor (Equation (1)), a nonzero phase term *α* can be introduced by tilting the illumination plane wave. In this way, the wavefront reaches different antennas with a phase delay. This results in a rotation of the array factor in each half-space above and below the array (see the animation in the Supplementary Material). The same principle is used in phased antenna arrays to achieve beam steering [24], [25].

**Figure 3:**
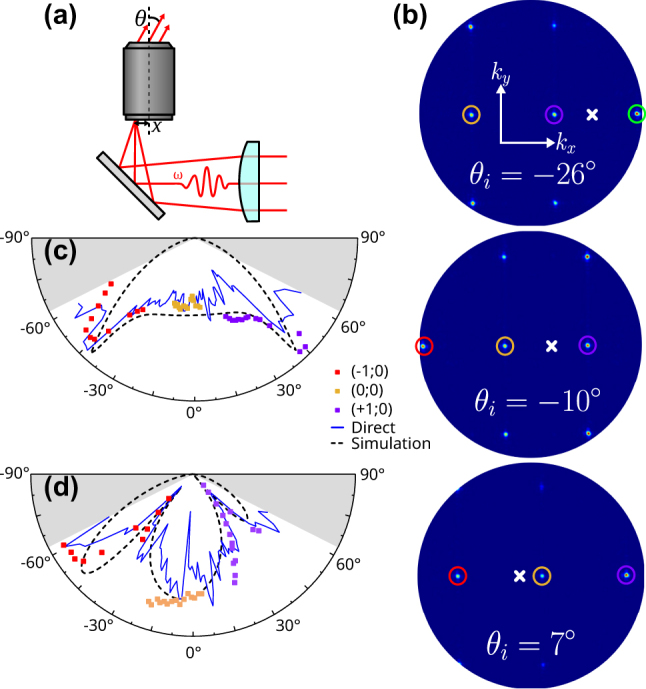
Second Harmonic emission pattern in air. (a) Sketch of the optical setup for tilted excitation. (b) Three representative BFP images at different incident angles, with *p*-polarized excitation. The (+2;0), (+1;0), (0;0), and (−1;0) are, respectively, circled in green, violet, orange, and red, and the angle of incidence is represented by a white cross. Normalized (c) *XZ* and (d) *YZ* cuts in the individual emission pattern, reconstructed from three diffraction orders, with experimental and simulated cuts in the BFP from Figure 1(c) and 2(c), respectively. The grayed areas represent the region inaccessible to the objective NA.

Thus, by controlling the direction of illumination, we can scan the position of the diffraction order in the BFP, sampling the single emitter far-field emission in different directions. In this way, the entire single emitter emission pattern can be reconstructed from separate measurements. We achieve tilted excitation by moving the focal point of the laser by Δ*x* on the back pupil of the objective (Figure 3(a)). The angle of incidence *θ*_i_ on the sample can then be calculated via Abbe’s sine condition *θ*_i_ = sin^−1^(Δ*x*/(*nf*)), with *n* being the refractive index of the immersion medium and *f* the focal length of the objective. Experimentally, we observe a translation of the SHG diffraction pattern in the back focal plane (Figure 3(b)). The emission angle *θ*_r_ corresponding to each spot can be calculated as *θ*_r_ = sin^−1^(NA/*n*). By integrating the SHG intensity of each diffraction point for different angles of incidence, the SHG emission pattern can be reconstructed. A similar technique was previously employed by Vercruysse et al. [27] (Figure S3), who used very sparse gratings with enough diffraction orders to obtain satisfactory insight into the single antenna’s behavior. However, their configuration was not very suitable for weak signals, which need contributions from many emitters to be workable.

**Figure 4:**
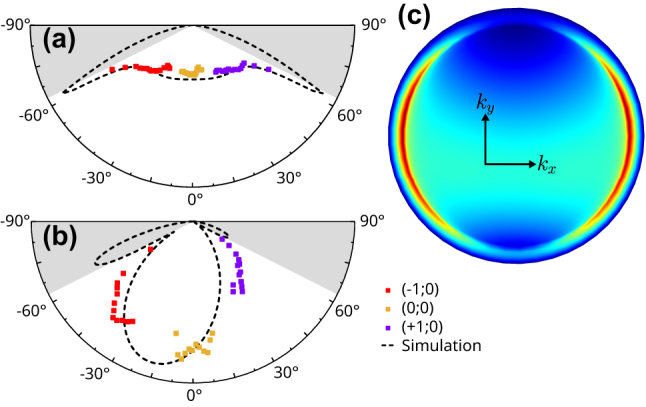
Second Harmonic emission pattern in water reconstructed (a) *XZ* and (b) *YZ* cuts in the individual emission pattern, with the antennas immersed in water. The grayed areas represent the uncollected light due to our limited NA. (c) Simulated BFP in the same situation.

Figure 4(c) and (d) show the power emitted at correspondence of different diffraction orders, sampling the *XZ* and *YZ* planes of emission, respectively. The cross-cuts of the experimental and simulated emission patterns (Figure 1(b) and (c)) have been added for comparison and an excellent agreement is obtained. While the most straightforward way to access the *YZ* plane would be a scan along *y* of the focus spot on the objective back pupil, for experimental convenience we employed a horizontal scan as for the *XZ* plane, rotating the sample and incident polarization by 90°.

Comparing with the direct BFP imaging of a single antenna, the reconstruction through sampling features lower noise, as we are exciting and collecting from numerous antennas, whose signal interferes constructively in the diffraction orders. Moreover, the greater number of emitters averages out individual effects such as particle dispersity, yielding a more stable result. Experimentally, while the acquisition of several BFP images is slightly longer than the direct imaging, focusing on the array is much simpler to achieve than on a single nanoantenna.

In addition, this technique is well suited to weak signals, such as SHG, especially when the nanostructures are immersed in a fluid, since the emission is reduced by the lower refractive index contrast. To illustrate the capabilities of our technique, we encapsulated the nanoantennas in a microfluidic channel. A PDMS (polydimethylsiloxane) channel was first fabricated by soft lithography with a width of 100 µm and height of 30 µm, using an already established protocol [28]. After oxygen plasma activation, the microchannel is bonded to the glass substrate by plasma activation, with the channel positioned above the arrays, and lastly connected to an inlet and outlet to allow immersing the structures in a liquid.

We again determine the nanoantenna emission pattern following the same procedure described above, while flowing deionized water in the channel. The resulting emission pattern is modified as a consequence of the different refractive index, with sharper lobes at an increased angle (Figure 4(c)), which lies outside the NA of our objective. Nevertheless, within the detection range, the measurement reproduces well the simulated emission pattern (Figure 4(a) and (b)).

## 4 Conclusions

We have simulated the second-harmonic emissive behavior of a grating of V-shaped antennas. After having experimentally validated the emission pattern, we have engineered a periodic grating of gold nanoantennas to optimize the emission of SHG radiation. In our case, the antennas have dipole-like emission pattern with a privileged radiation direction at the critical angle of the sub- and superstrate interface. Arranging the array period so that one of the diffraction orders overlaps with this direction yields a sizable increase of the collected signal. This optimization is directly applicable to structures with similar resonant features, and more broadly, to any system provided the emission pattern is known. This work highlights the relevance of considering the coupling of antenna and grating modes when designing arrays for maximal light emission, which is frequently ignored in sub-wavelength metasurface designs.

Furthermore, we have demonstrated a method to experimentally reconstruct the single emitter radiation pattern from the collective array response, by rotating the grating diffraction pattern to sample different positions on the BFP. This method is simple to realize experimentally and well suited to study weak signals while possessing a high signal-to-noise ratio and independence from particle irregularities, as the light is collected from numerous emitters. Finally, as illustrated in the last part, the emission pattern of dipole antennas is very sensitive to refractive index changes and features a sharp angular width, making it a potential tool for sensing purposes [28].

## Supplementary Material

Supplementary Material Details
