# Supplementary material for: Far-field mapping and efficient beaming of second harmonic by a plasmonic metagrating
Source: Nanophotonics. 2024 Apr 17;13(18):3609–14. doi: 10.1515/nanoph-2023-0842 (PMC11501601; doi:10.1515/nanoph-2023-0842)
Supplement: Supplementary file 1 — Supplementary Material Details [file j_nanoph-2023-0842_suppl_001.pdf]

Supplementary Information:
Far-field mapping and efficient beaming of Second Harmonic by a plasmonic metagrating

Augustin Verneuil[1,2], Agostino di Francescantonio[2], Attilio Zilli[2], Julien Proust[1], Daniela Petti[2], Marco Finazzi[2], Michele Celebrano[2], and Anne-Laure Baudrion[1]

[1]Light, Nanomaterials, Nanotechnologies, Université de Technologie de Troyes, Troyes, France
[2]Dipartimento di Fisica, Politecnico di Milano, Milan, Italy

Contents

A Simulated near-field distributions **1**
a Simulation process 1
b Near field distributions 3

B Study of the SLR coupling regime **4**

C Near-infrared extinction spectra **5**

D Optical setup for nonlinear characterization **6**

E Upconversion efficiency **7**

F Power study **8**

A Simulated near-field distributions

a Simulation process

General considerations The model consists of a single V-shaped antenna, placed on a dielectric substrate of glass. The antenna edges have been rounded to reproduce better the fabricated geometry, and avoid local field singularities. Finally, the whole model is surrounded in Perfectly Matched Layers (PML), absorbing the outgoing electric field to act as infinite free space. (fig. S1a) The exciting field at the fundamental wavelength (1550 nm) is a plane wave incident from the substrate half-space.

Meshing The whole model was meshed with tetrahedrons, except for the PML. The latter used quadrilateral elements, ensuring they would face the particle out of spurious reflection concerns.

The maximal element size inside the particle was kept under 12 nm for the simulation at the fundamental, and 6 nm at the harmonic wavelength. The meshing for each arm is mirrored to avoid any dissymmetry issue.

The rest of the volume was limited to $\lambda/5$ (adjusted for the refractive index of the medium). (fig. S1b)

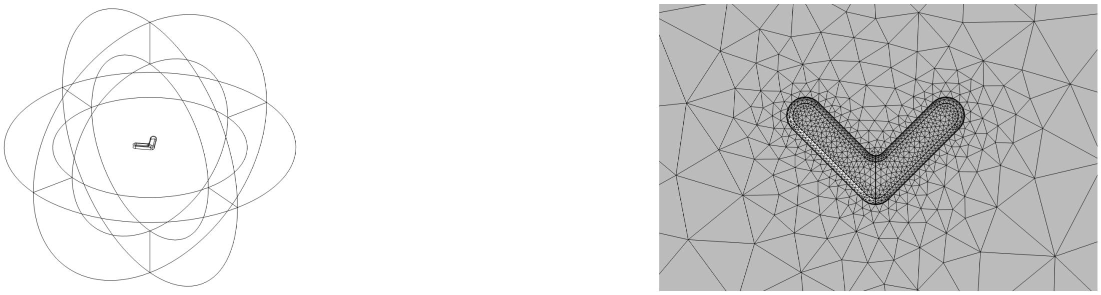

(a) Model domains, including the PML (b) Model meshing, detail for the antenna

Figure S1

Nonlinear current sources We want to simulate the nonlinear current sources on the surface of the antenna, as those are the most significant contribution to the overall SHG emission.[1] Since COMSOL does not implement a suitable boundary condition to represent currents oriented along the normal of the surface, we can use a weak form contribution, that will constrain the solving of the wave equation for electric fields:

$$\nabla \times \mu^{-1} \nabla \times \mathbf{E} + (i\omega\sigma - \omega^2\varepsilon)\mathbf{E} = 0 \tag{1}$$

We consider time-harmonic fields in the $+i\omega t$ convention used by COMSOL. An additional nonlinear current term is added to the Mawxell-Ampère law:

$$\nabla \times \mathbf{H} = \varepsilon \frac{\partial \mathbf{E}}{\partial t} + \mathbf{J} + \mathbf{J}_{NL} = i\omega\varepsilon\mathbf{E} + \sigma\mathbf{E} + \mathbf{J}_{NL} \tag{2}$$

We take the curl of the Maxwell-Faraday law, and replace with eq. (2):

$$\nabla \times \mathbf{E} = -i\omega\mu\mathbf{H} \tag{3}$$

$$\nabla \times \left(\mu^{-1}\nabla \times \mathbf{E}\right) = -i\omega\nabla \times \mathbf{H} \tag{4}$$

$$= -i\omega\left[\left(i\omega\varepsilon + \sigma\right)\mathbf{E} + \mathbf{J}_{NL}\right] \tag{5}$$

$$\nabla \times \left(\mu^{-1}\nabla \times \mathbf{E}\right) + \left(i\omega\sigma - \omega^2\varepsilon\right)\mathbf{E} = -i\omega\mathbf{J}_{NL} = \omega^2\mathbf{P}_{NL} \qquad \text{since } \mathbf{J}_{NL} = \frac{\partial \mathbf{P}_{NL}}{\partial t} \tag{6}$$

Then, to obtain the weak form of eq. (6), we multiply by the test function $\overline{\mathbf{E}}$ which locally samples the solution and integrate over the simulation domain Ω:

$$\int_\Omega \left[\nabla \times \left(\mu^{-1}\nabla \times \mathbf{E}\right) + \left(i\omega\sigma - \omega^2\varepsilon\right)\mathbf{E}\right] \cdot \overline{\mathbf{E}} = \int_\Omega \omega^2\mathbf{P}_{NL} \cdot \overline{\mathbf{E}} \tag{7}$$

We recognize the left-hand member of the wave equation eq. (1) inside the left integral. Thus, the weak contribution implementing the nonlinear current is given by the right-hand side, which can be further relaxed to a surface integral, as the charges lie on the interface Σ of the particle:

$$\int_\Sigma \omega^2\mathbf{P}_{NL} \cdot \overline{\mathbf{E}} \tag{8}$$

The nonlinear polarization is obtained from the fundamental electric field: $\mathbf{P}_{NL} = \varepsilon_0 \overleftrightarrow{\chi}^{(2)} \mathbf{E}^2$, and since the most prominent component of the susceptibility tensor is $\chi^{(2)}_{\perp\perp\perp}$,[2] we can write: (using $\hat{\mathbf{n}}$ as a unit vector normal to the surface of the antenna)

$$\omega^2 \mathbf{P}_{NL} \cdot \overline{\mathbf{E}} = \omega^2 \varepsilon_0 \chi^{(2)}_{\perp\perp\perp} (\hat{\mathbf{n}} \cdot \mathbf{E}^2_{\perp}) \cdot \overline{\mathbf{E}} \tag{9}$$

$$= \omega^2 \varepsilon_0 \chi^{(2)}_{\perp\perp\perp} \begin{bmatrix} n_x (n_x E_x + n_y E_y + n_z E_z)^2 \\ n_y (n_x E_x + n_y E_y + n_z E_z)^2 \\ n_z (n_x E_x + n_y E_y + n_z E_z)^2 \end{bmatrix} \cdot \begin{bmatrix} \overline{E_x} \\ \overline{E_y} \\ \overline{E_z} \end{bmatrix} \tag{10}$$

$$= \omega^2 \varepsilon_0 \chi^{(2)}_{\perp\perp\perp} (n_x E_x + n_y E_y + n_z E_z)^2 (n_x \overline{E_x} + n_y \overline{E_y} + n_z \overline{E_z}) \tag{11}$$

b Near field distributions

Below are given the near-field distributions solved within COMSOL, at the fundamental and harmonic wavelengths. The excitation at the fundamental (fig. S2a) is polarized along the arms of the particle (ie. horizontally on the image below), and the resulting field enhancement is plotted. Then, nonlinear current sources are calculated and a second simulation step yields the field distribution at the harmonic wavelength (fig. S2b). It should be noted that the harmonic simulation step (fig. S2b) is not quantitative.

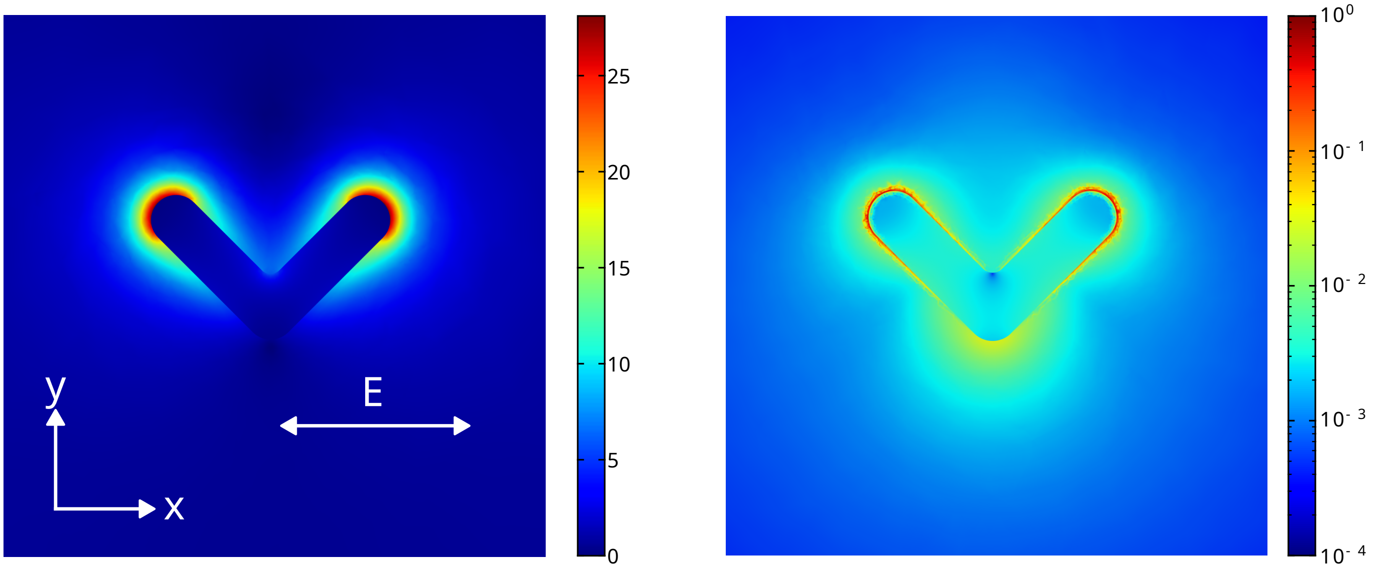

(a) Field enhancement ($|E|_{FW} / E_0$) at the fundamental wavelength

(b) Normalized near field ($|E|_{SH} / |E|^{\max}_{SH}$) at the harmonic wavelength (logarithmic color scale)

Figure S2

B Study of the SLR coupling regime

The decoupling of the antenna emission with the array factor, discussed in the main text, is valid in the absence of interactions between the individual emitters. Thus, the influence of SLRs on the SH must be shown to be negligible.

Firstly, index matching is an important criterion for the array to support collective modes.[3, 4] Air/BK7 ($\Delta n = 0.511$ at $\lambda_{SH} = 775$ nm) and water/BK7 ($\Delta n = 0.183$ at λ_{SH}) are both significant index discrepancies that would hinder potential SLRs. Secondly, as shown in the paper text, the SH emission corresponds to that of a vertical dipole (see fig. S3a), ie. very directional along the horizontal x axis. Thus any SLR mode along a diagonal or the vertical y axis is expected to be inefficient.[5]

To further investigate the decoupling of the antenna and array factors, we plot the position of the Rayleigh anomalies (fig. S3b), using the following expression derived from the Laue condition:

$$\mathbf{k}_G = \mathbf{k}_\parallel + \mathbf{K} \tag{12}$$

$$2\frac{2\pi n_m^{SH}}{\lambda_{FW}} = 2\frac{2\pi n_s^{FW}}{\lambda_{FW}} \sin\theta_i \pm i\frac{2\pi}{a} \tag{13}$$

$$a = i\frac{\lambda^{FW}}{2(n_m^{SH} \pm n_s^{FW} \sin\theta_i)} \tag{14}$$

with a being the period satisfying the RA condition, $i \in \mathbb{N}^*$ an integer corresponding to the diffraction order, a the pitch of the grating, λ^{FW} the fundamental wavelength, θ_i the incident angle, and n_s^{FW}, n_m^{SH} respectively the refractive indices of the substrate at the fundamental wavelength, and of the propagation medium of the in-plane diffraction order at the harmonic wavelength.

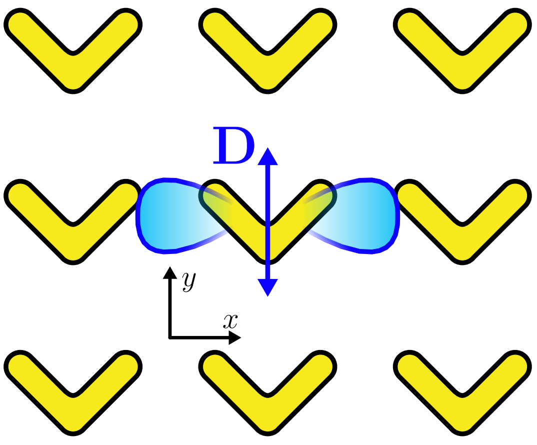

(a) Schematic of the SH dipole **D** and its associated emission within the array

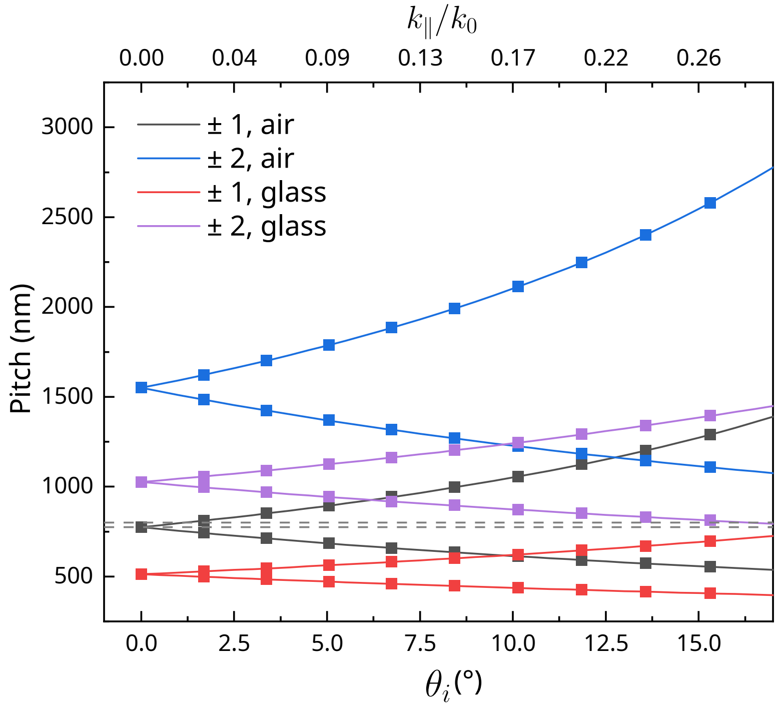

(b) Periods supporting an SLR corresponding to ±1 and ±2 ($\pm i$ in eq. (14)) diffraction orders in the sub- or superstrate as function of the incident angle. Data points are placed at the studied angles. Dashed horizontal lines at $a = 800$ and 775 nm represent the X and Y periods of the grating used during the radiation pattern reconstruction.

Figure S3

From fig. S3b we find that the only two situations in the paper supporting LSRs are when crossing a first-order diffracted beam in air, close to normal incidence ($\theta_i = 0°$ and $1.18°$ for $a = 775$ and 800 nm, respectively). The first of these two conditions leads to a SLR oriented along y in fig. S3a, expected to be inefficient.

We then perform numerical simulations to ensure that the emission behavior was not significantly affected under these conditions. To be able to simulate potential SLRs, the spherical simulation domain described in section A is replaced by a rectangular slab, with periodic boundary conditions on each side to mimick an infinite array (see fig. S4a). We then study the Trans- and Epi-collected SH power (ie. emitted in the air and glass half-spaces, respectively), as well as the power absorbed by the antenna at the two possible SLR angles (0° and 1.18°), along with a reference at 5°:

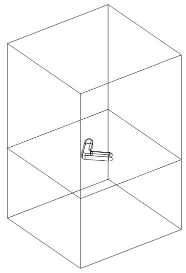

(a) Simulation domain

Incident angle (°)	Trans (a.u.)	Epi (a.u.)	Absorbed (a.u.)
0.00	0.4732	5.0077	1.9366
1.18	0.4732	5.0075	1.9368
5.00	0.4731	5.0074	1.9370

(b) Trans-, Epi-collected and absorbed SH power at different incident angles (note that these simulated power values are not quantitative)

Figure S4

We find from fig. S4b that the values of emitted SH power are negligibly affected by the presence of SLRs (at $\theta_i = 0°$ and $1.18°$), comparing to a situation incompatible with a collective mode ($\theta_i = 5°$), since the values of powers agree within 5 significant digits. We finally conclude that the SLRs supported by our grating do not significantly influence their SH emission, and thus the decoupling of the antenna and array emission patterns is valid.

C Near-infrared extinction spectra

Setups The plasmon resonances at the fundamental and harmonic wavelength are characterized by NIR spectroscopy, for several of the arrays fabricated. The former range (figs. S5a and S6b) was investigated on an Andor Kymera 328i spectrometer, with a pinhole in an intermediary focal plane. For the latter (figs. S5b and S6a), we used a cryogenically cooled Teledyne PyLoN-IR, with spatial filtering achieved using the spectrometer slit and its single pixel line.

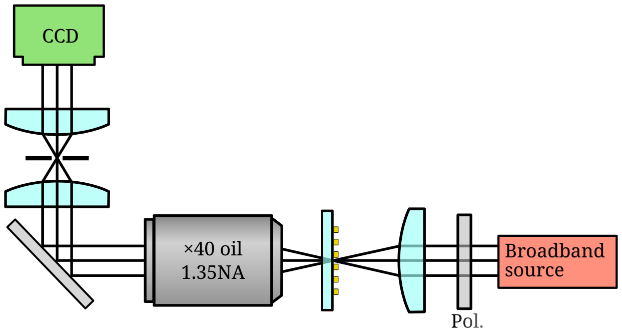

(a) Extinction setup for the fundamental resonance

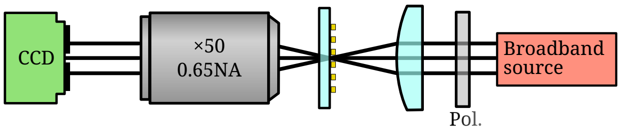

(b) Extinction setup for the harmonic resonance

Figure S5

Results The decrease in extinction intensity as the pitch increases can be explained by the diminishing density in antennas, while the slight redshift in center wavelength is due to proximity effects during the e-beam lithography fabrication step, which affect the fabricated geometries.

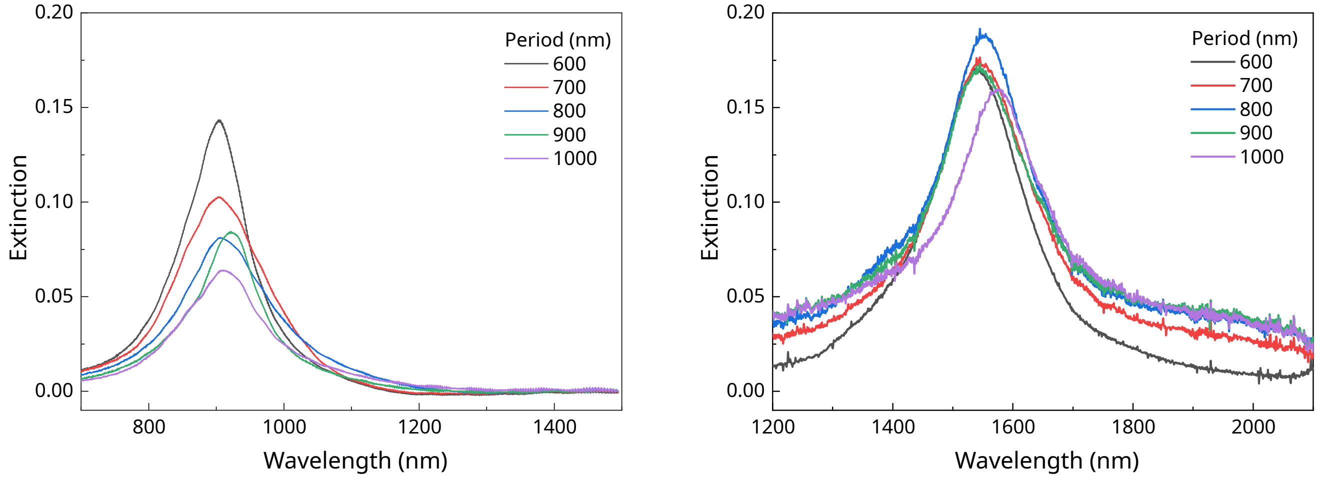

(a) Spectra for the harmonic resonance (b) Spectra for the fundamental resonance

Figure S6

D Optical setup for nonlinear characterization

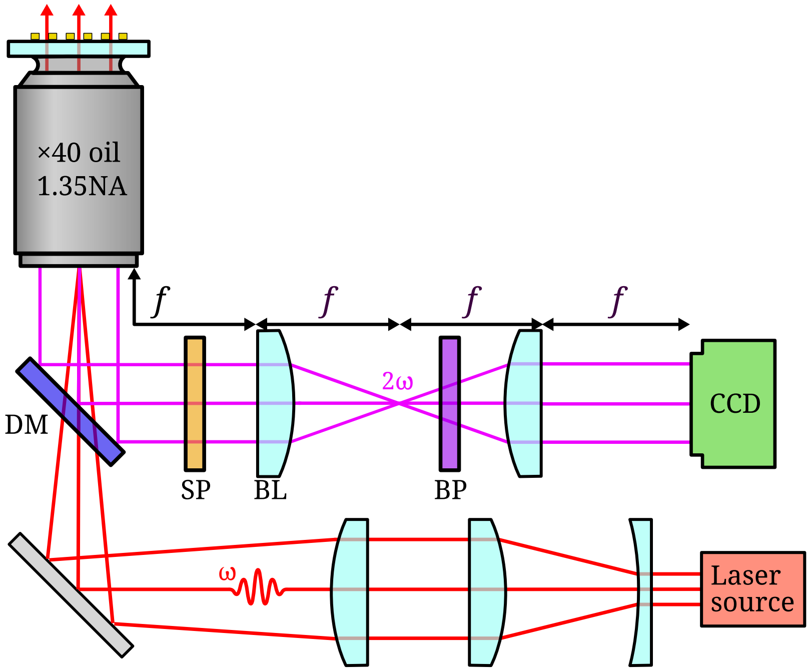

Figure S7: Simplified sketch of the optical setup for nonlinear measurements. DM: Dichroic mirror, SP: Short-pass filter, BP: Band-pass filter, BL: Bertrand lens.

In the excitation path, the incoming laser beam (OneFive, Origami 15-80) is first expanded to better fill the subsequent lens, then focused on the back pupil of the objective. The resulting light at the aperture of the objective is thus nearly collimated. For the single particle BFP measurement, this lens is removed.

In the collection path, reflected fundamental light is first rejected by the dichroic mirror, then by a succession of short-pass filtera (Thorlabs FESH1000, Thorlabs FESH800). Eventual fluorescence is then excluded by a band-pass filter around the SH wavelength (Omega 775AF25).

The BFP is imaged onto a CCD camera (Andor iKon) by means of a 4-f arrangement. The Bertrand lens can be flipped out of the detection path to switch between real and Fourier space imaging modes.

E Upconversion efficiency

Following the procedure highlighted by Celebrano et al. [2], we calculate the upconversion efficiency from the detected SH counts on the CCD camera, adjusting for the transmittance of the optical elements in the collection path, as well as the quantum efficiency and sensitivity of the CCD.

Parameter	Wave-length (nm)	Laser rep. rate (MHz)	Laser pulse duration (fs)	Average power (mW)	Spot diameter (µm)	Metasur-face size (µm)	Peak power (W)
Value	1550	80	160	0.48	30	20	37.5

Table S1: Quantities characterizing the excitation of the metasurface

Parameter	SH collected photons (cts/s)	SHG power (W)	SHG peak power (W)	Max. conversion efficiency	Peak nonlinear coefficient (W^{-1})
Value	1.4×10^{6}	3.7×10^{-14}	2.9×10^{-9}	7.7×10^{-11}	2.0^{-12}

Table S2: Quantities characterizing the conversion efficiency the metasurface

F Power study

The dependence of the collected signal as a function of the exciting power is studied. SH counts are measured in three diffraction orders. The counts are then converted to a peak instant power using the above table.

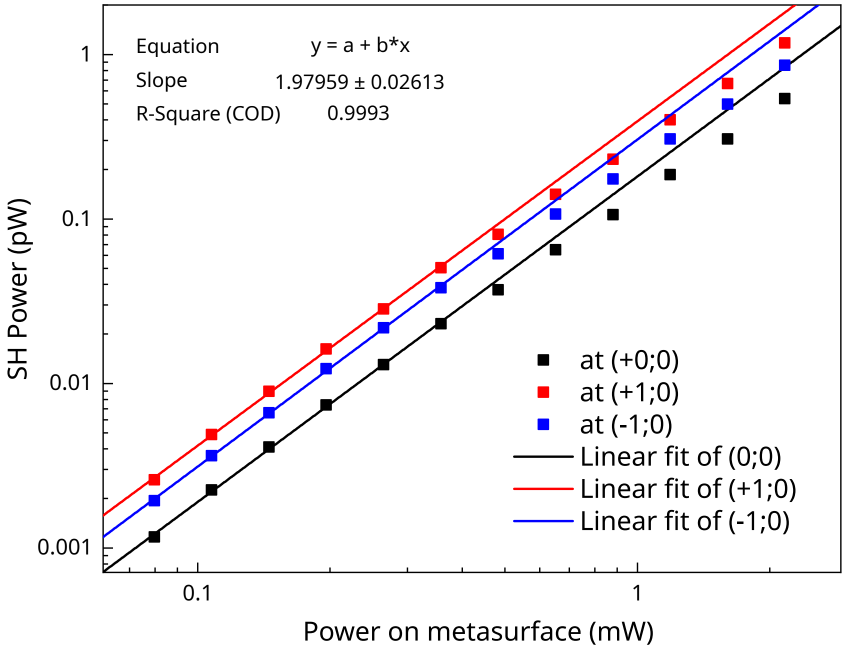

Figure S8: Power study

From a linear fit on a log-log plot, a power law of 2 is obtained, confirming that the observed photons are produced by a second-order nonlinear process. The divergence above 0.5 mW can be explained by a saturation of the process, or photodamage. Those point were excluded from the fitted data.

References

[1] G. Bachelier et al. "Origin of optical second-harmonic generation in spherical gold nanoparticles: Local surface and nonlocal bulk contributions". In: *Physical Review B* 82.23 (2010), p. 235403. DOI: 10.1103/PhysRevB.82.235403.

[2] Michele Celebrano et al. "Mode matching in multiresonant plasmonic nanoantennas for enhanced second harmonic generation". In: *Nature Nanotechnology* 10.5 (2015), pp. 412–417. DOI: 10.1038/nnano.2015.69.

[3] V. G. Kravets et al. "Plasmonic Surface Lattice Resonances: A Review of Properties and Applications". In: *Chemical Reviews* 118.12 (2018), pp. 5912–5951. DOI: 10.1021/acs.chemrev.8b00243.

[4] Anton D. Utyushev, Vadim I. Zakomirnyi, and Ilia L. Rasskazov. "Collective lattice resonances: Plasmonics and beyond". In: *Reviews in Physics* 6 (2021), p. 100051. ISSN: 24054283. DOI: 10.1016/j.revip.2021.100051.

[5] Mikko J. Huttunen et al. "Ultra-strong polarization dependence of surface lattice resonances with out-of-plane plasmon oscillations". In: *Optics Express* 24.25 (2016), p. 28279. DOI: 10.1364/OE.24.028279.
